# A qualitative exploration of interactional and organizational determinants of collaboration in cancer palliative care settings: Family members’, health care professionals’ and key informants’ perspectives

**DOI:** 10.1371/journal.pone.0256965

**Published:** 2021-10-06

**Authors:** Marco Bennardi, Nicola Diviani, Georg Stüssi, Piercarlo Saletti, Claudia Gamondi, Ivan Cinesi, Sara Rubinelli

**Affiliations:** 1 Person-centered Healthcare & Health Communication, Swiss Paraplegic Research, Nottwil, Canton of Lucerne, Switzerland; 2 Department of Health Sciences and Medicine, University of Lucerne, Lucerne, Canton of Lucerne, Switzerland; 3 Hematology, Oncology Institute of Southern Switzerland, Ospedale San Giovanni, Bellinzona, Canton of Ticino, Switzerland; 4 Clinica Luganese Moncucco, Lugano, Canton of Ticino, Switzerland; 5 Palliative Care, Oncology Institute of Southern Switzerland, Ospedale San Giovanni, Bellinzona, Canton of Ticino, Switzerland; 6 Palliative TI–Associazione Cure Palliative Ticino, Cadenazzo, Canton of Ticino, Switzerland; Deakin University, AUSTRALIA

## Abstract

As life expectancy has increased, a growing number of people experience conditions, including cancers, that carry complex health and social needs. Palliative care services have the potential to address these needs but face significant obstacles. One major obstacle is suboptimal interprofessional collaboration. This study’s goal was an in-depth exploration of interactional and organizational barriers and supports of collaboration in palliative care in Switzerland. We sought the perspectives of health care professionals, patients’ family members and leaders and experts in oncology/palliative care delivery (key informants) through interviews and focus groups with fifty HPs and key informants and ten patients’ family members. Qualitative analyses of interviews and focus groups used framework analysis. We identified three major themes of interaction: personal characteristics, communication, and connectedness with other health care professionals; and three major organizational themes: service characteristics, standardized communication and processes, and service coordination and promotion. Based on our findings, we recommend that health care professionals consider strategies to increase their collaboration and communication skills and opportunities to interact. We advocate the implementation of methods for coordinating services, standardization of consultation/referral procedures and communication between health care professionals, and the promotion of underutilized services to foster successful, sustainable collaboration.

## Introduction

Life expectancy has increased considerably in recent decades [1] and is predicted to continue increasing in industrialized countries [2]. However, with a growing aging population, the prevalence of disorders connected to aging is also rising. As a consequence, a growing number of patients, including those suffering from cancers, have multiple health and social needs [3].

Oncology patients often have significant symptom burden, including dyspnea, nausea, fatigue and pain, which can cause distress and decrease their quality of life [4]. Patients provided with early palliative care (PC) can experience relief of symptoms and improvements in quality of life, satisfaction, mood, resource use and advanced care planning [4]. Optimal integration of PC into health care systems has not yet been achieved; PC services are not provided to all in need in most countries, even in high-resource systems [5]. Many barriers to and facilitators of the implementation of PC services have been identified. At the individual level these can be related to the roles of patients and health professionals (HPs) [6]. Among the main facilitators, effective collaboration between HPs has consistently been shown to play a central role [7–9]. In this context, a major barrier is an unfavorable attitude towards collaboration [6]. This barrier can be a consequence of poor communication skills [8] or the lack of a culture of engaging in collaboration [10]. These can arise from personal reasons [11], organizational culture, or resource constraints [8], all of which may encourage working alone and discourage cooperation.

Previous work has not systematically addressed the interactional and organizational aspects behind the lack of collaboration in PC settings for cancer patients. To identify the aspects impeding a successful collaboration, it is essential to listen to the voices of HPs, who are primarily responsible for the decision of whether to collaborate (e.g. consult a PC team)—but also to the voices of leaders and experts in oncology and PC delivery (key informants, KIs), who may have a more comprehensive understanding of the situation of cancer PC and of collaborative processes. KIs can play an extremely relevant role in deciding which services to invest in and may be able to shape policy. Finally, listening to patients is vital, though this point of view has been largely left out of previous studies of barriers to and facilitators of PC utilization [6].

To best of our knowledge, no previous explorative studies on collaboration in oncology and PC including the perspective of different actors involved in health care delivery and utilization have been conducted. Previous studies in different patient care settings, however, have reported that interactional and organizational aspects are among the main barriers to collaboration [12,13]. Therefore, our objective was to investigate the experience of interprofessional collaboration between HPs working with oncology patients in primary, oncology and PC settings. The focus was on the identification of interactional and organizational barriers and facilitators to successful interprofessional collaboration within (e.g. within PC) and between medical specialties (e.g. between oncology and PC).

## Methods

Given the current limited understanding of the role of collaboration in PC and oncology settings in Switzerland, an exploratory qualitative approach was selected to investigate the perceptions and experiences of interprofessional collaboration from the perspective of HPs, KIs and patients’ family members in the Italian-speaking part of Switzerland.

### 1.1. Setting, sample and recruitment

Our sample comprised HPs (individuals working in direct contact with patients, such as medical doctors, nurses, psychologists, spiritual assistants, social workers, and volunteers), KIs (people active in PC at the policy level), as well as family members of recently deceased cancer patients. Our sample of HPs included professionals employed in primary, oncology and PC settings to provide a comprehensive picture of interprofessional collaborative processes. Participants were employed by four public hospitals, two private clinics, eight organizations and seven care practices. For analytical purposes, we categorized as PC providers all participants with a specialization in PC, for practitioners only, and for the other HPs those nurses, psychologists, spiritual assistants, social workers who worked at least 80% for specialized PC service or were leaders or experts (KI) or volunteers in PC. We categorized as non-palliative-care (NPC) providers all participants who did not belong to a specialized PC team (e.g. general practitioners (GPs) and oncology providers). Recruitment of HPs and KIs was performed through a variety of channels, from formal invitations via professional organizations to informal snowball sampling techniques. Family members were recruited through leaflets distributed in pharmacies and in the local hospital’s PC unit. Additionally, we asked the two main local home health care services and the local cancer league, which organize self-help groups for family members of deceased cancer patients, to refer family members to us.

### 1.2. Data collection

Interviews with HPs and KI were conducted between August 2018 and January 2019, while those with family members were conducted in January and February 2020. Interviews were conducted one-on-one, in a private space in each hospital/clinic/organization, or via telephone when an in-person interview could not be arranged (n = 2). HPs and KIs were interviewed individually using a semi-structured interviewed. Family members of deceased patients were interviewed either as part of a focus-group discussion or using a semi-structured individual interview. Some family members preferred to tell their experiences individually, due to the sensitivity of the topic (e.g. they still felt very emotionally involved talking about the end of life of their loved ones or felt more comfortable to tell their experiences with health care services in a private environment). The patients’ deaths occurred from 12 to 24 months prior to the interviews or focus groups. Researchers in the health sciences with extensive experience in qualitative methodology conducted all interviews and facilitated the focus group.

We developed a semi-structured interview guide (S1) and a focus-group interview guide (S2) with topics derived from the literature on interprofessional collaboration and communication in health care (Box 1) [12–14]. The guides have a narrative approach, and perceptions and experiences related to interprofessional collaboration were evoked by asking HPs/KIs questions. Patients’ family members were asked about their experience with health care services, and also asked what aspects related to collaboration within services they observed (e.g. if they perceived conflicts, how they perceived HPs’ roles). For instance, family members were asked, *Did your main care providers suggest additional services*? *How did you perceive the collaboration between these services/health providers*? *Would you say that there were many services*? *Were health professionals’ roles clear to you and to your loved one*? The use of semi-structured interview and focus group guides enabled flexible data collection, allowing the opportunity to elicit open responses, while ensuring relevant topics were covered in each interview or focus group [15]. Participants were encouraged to open new directions in the discussion or interview [16]. The first author (MB) carried out five pilot interviews and a focus-group pilot interview to guarantee the appropriateness of questions to gather information relevant to the aim of the study.

**Box 1. Topics covered in interviews with health care professionals and key informants and sample questions**

Willingness to collaborate (e.g. openness to collaboration, helpfulness, expectations from collaboration, being accustomed to working on a team, common goals)

*Examples: In general*, *what is your experience with collaboration? What role does collaboration between health providers from different services play in oncology patients’ care? Do you have examples in which you or your colleagues worked with other services’ providers?*

Trust in others (e.g. trust in others’ abilities, in other professionals) and mutual respect (e.g. of others’ skills and knowledge)

*Example: How are important other providers*’ *competences to the provision of high-quality patient care?*

Interpersonal communication (e.g. mutual communication, direct connection, active listening)

*Examples: Have you experienced*, *or heard about from colleagues*, *critical situations related to communication between health providers? Or between different organizations?*

Organization’s philosophy and administrative support for collaboration (e.g. leaders motivating HPs to adopt collaborative practices, climate of openness towards collaboration)

*Examples: What aspects within your organization support collaboration between professionals? What*, *in particular*, *facilitates early consultation with PC services?*

Team resources (possible resources provided by the employer for collaboration, e.g. time to interact or space to meet with other providers).

*Examples: Are there factors in your health care organizations that support or impede collaboration?* *In other organizations of which you are aware?*

Management and communication processes established by the organization (standards, policies, protocols, e.g. type of mechanisms available to exchange information, division of work and common rules)

*Examples: How does collaboration usually occur within the organization? For instance*, *could you describe the steps oncology providers or general practitioners take before referral to*, *or consultation with*, *a PC team?*

### 1.3. Data analysis

Interviews and focus groups were audio-recorded and transcribed by the authors for framework analysis [17,18].

After repeated reading of the transcripts, the authors worked together to identify a thematic framework. The thematic framework was devised and refined on the basis of the research question, notes taken during interviews, and implicit connections between ideas [17]. Several methods of refining the thematic framework were adopted at subsequent stages of analysis to ensure the original research question was being fully addressed. The analysis process followed both a priori research inquiries and a priori issues related to the dynamics of collaboration from previous studies of collaboration, which were integrated into a unique model by creating a framework matrix [12–14,19]. We maintained open minds regarding potential additional issues emerging from the data [20]. The themes were generated from the data set by reviewing the matrix and making connections within and between individual views and experiences, and categories of the matrix [19,20]. We also considered topics, ideas and patterns of meaning that appeared repeatedly. Finally, we performed data mapping and interpretation [17].

Based on the categorization proposed by San Martín-Rodríguez et al. [13], we considered barriers and facilitators at the *interactional* and *organizational* levels in the analysis. The *interactional* level includes all aspects related to interpersonal relationships between HPs, while the *organizational* level includes all aspects of an organization or a discipline, that facilitate or hinder interprofessional collaboration.

### 1.4. Ethical considerations

Approval to conduct this study was granted in June and July 2018 by the ethics committees of three regions of Switzerland (Canton Ticino, Canton Grisons and central Switzerland). Participants were provided with a study information sheet upon enrollment and asked to sign a consent form.

## Results

The total sample was 60 participants. Of these, 40 were HPs (general practitioners, specialized practitioners, psychologists, nurses, social workers, spiritual assistants, volunteers). All had prior experience with patients requiring oncological care and/or PC. The sample also comprised 10 KIs—including heads of oncology and PC organizations (e.g. professional societies, non-profit organizations) and institutions (e.g. clinics) and experts in the aforementioned medical fields—and 10 family members of deceased oncology patients (see Tables 1 and 2). Among health care professionals and key informants, 15 worked in palliative care and 35 worked in primary care or oncology. There were 67% of PC providers who were female compared to only 46% of NPC providers. In total, 33% of PC providers had received their training in Switzerland, compared to 77% of NPC providers. Overall, the vast majority of participants (94%) were senior professionals with 11 or more years of professional experience. Interviews lasted 45–75 minutes, and the focus group 120 minutes.

**Table 1:** Participants’ characteristics.

Characteristic	Total	Health Care Professionals	Key Informants	Family Members
Total n	60	40	10	10
Gender n				
**Female**	35	23	4	8
**Male**	25	17	6	2
Age (years) n				
**18–30**	1	1	0	0
**31–40**	7	6	1	0
**41–50**	16	14	0	2
**51–60**	19	13	3	3
**≥61**	17	6	6	5

**Table 2:** Health care providers education and career stage.

Characteristic	Total	Health Care Professionals (HPs)				
		GPs	Specialised Doctors	Nurses	Psychologists	Social workers, spiritual assistants, volunteers
	**40**	**6**	**10**	**10**	**6**	**8**
Main education n (HPs/KI)						
**Switzerland**	24	5	5	6	2	6
**Abroad**	16	1	5	4	4	2
Career stage n (HPs/KI)						
**Junior (1–10 years)**	9	0	1	4	0	4
**Senior (≥11 years)**	31	6	9	6	6	4

**Fig 1:**
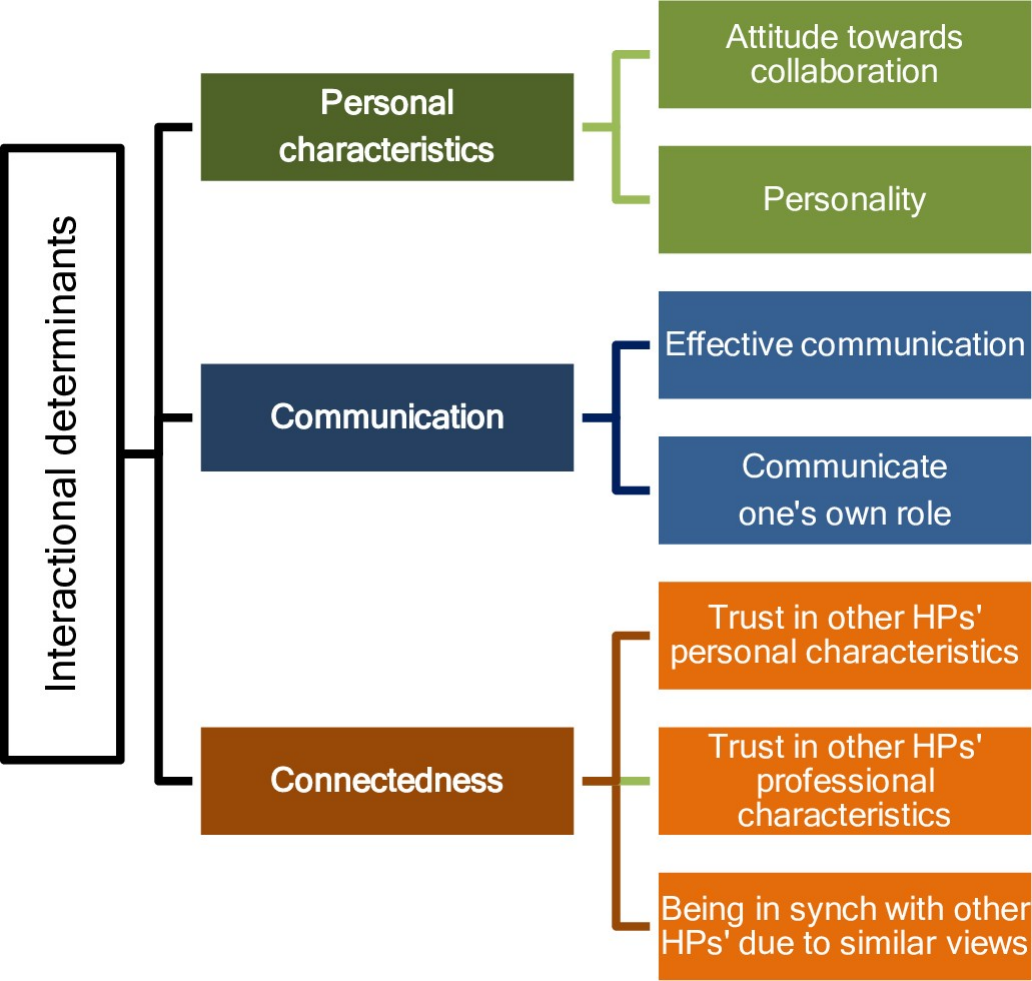
Themes summarizing interactional determinants to collaboration.

**Fig 2:**
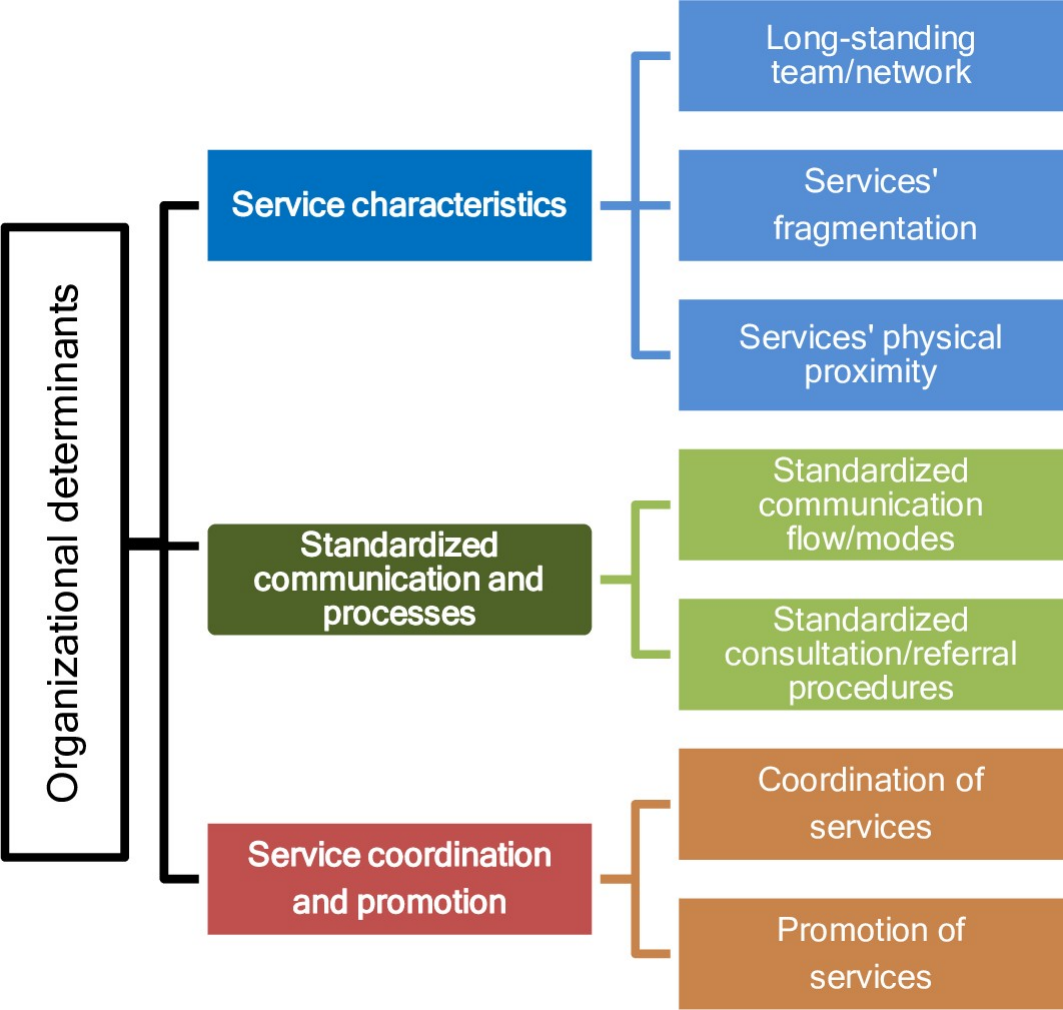
Themes summarizing organizational determinants to collaboration.

We identified six themes (Figs 1 and 2) that describe barriers and facilitators to successful collaboration, and cover aspects linked to both interprofessional (e.g. within a PC team) and interdisciplinary (between oncologists and PC providers) collaboration. These themes were organized into two main areas—interactional and organizational barriers and facilitators.

### 1.5. Interactional barriers to and facilitators of successful collaboration

Interactional barriers and facilitators include all factors related to interactions between HPs, within teams or between professionals working for different services. Analysis revealed three themes related to these factors (Fig 1 and Table 3).

**Table 3:** Interactional barriers and facilitators to successful collaboration from perspectives of PC and NPC providers, patient family members, and illustrative data extracts.

Theme/Subthemes	Illustrative quotes	
**Personal characteristics:** • Attitude towards collaboration • Personality	*Sometimes I get to ask other HPs questions*, *and sometimes they don’t reply […] you are going to look elsewhere*, *but you’re still thinking*, *"why aren’t you willing to reply…*? *Do you find me so unlikable*?*” At the end of the day*, *we are all in the same boat… you should give me the brush-off instead*, *and stop… so that I would know what you think*. (PC-Physician, ID2).	*I repeat*, *here we are privileged because oncology patients’ access to PC is planned*, *but this can occur also instantly*, *thanks to PC team members’ extreme helpfulness and the fact that they are based on the same floor we are* (NPC-Physician,ID11).
**Communication:** • Effective communication • Communicate one’s own role	*There is a communication today*, *I would say very effective*, *certainly from colleagues (of the PC services) as far as I am concerned*. *There is an involvement*, *a maintenance of the attention on the patient*, *the colleagues inform me*, *almost daily*, *of what is happening*. *Therefore*, *we work together with a mood that seems to me directed to be effective*. (NPC-Oncologist, ID11).	*But it is necessary that the physician activates his own antennas in the direction of what other HPs do*, *it must be a mutual thing*, *a two-pronged thing… because just waiting to receive information is not particularly interesting…* (NPC-Physician,ID13).
**Connectedness** • Trust in other HPs’ personal characteristics • Trust in other HPs’ professional characteristics • Being in synch with other HPs’ due to similar views	*The more the mutual knowledge*, *the more the mutual trust in the [HPs’] ability to take care of the patient and so… […]So*, *mutual trust between HPs compensates for this point*, *encouraging and facilitating collaboration* (NPC-Key Informant,ID20).	*The physician has to know that he can count on a service which works well… and to have trust in it… this encourages collaboration* (PC-Psychologist,ID26).

#### 1.5.1. Personal characteristics

A number of characteristics related to HPs’ personalities and attitudes, such as openness to collaboration or agreeableness, play a crucial role in collaboration. For instance, participants reported that lack of a positive attitude toward collaboration limits the chance to engage in teamwork. They highlighted that a negative attitude toward teamwork might be due to unfamiliarity. In addition to attitude, some specific personality traits were considered incompatible with teamwork. Presumption, for instance, was mentioned as a major obstacle, especially when providers who refer patients to other services (referring providers), hear from those who supply the services (rendering providers), that they can address certain issues (such as pain) better. Some referring providers reported that this situation makes them feel uncomfortable and, therefore, makes them less willing to collaborate. Nevertheless, some of the providers to whom the patient is referred are aware of this:

*The lack of collaboration is one’s own fault*, *is related to how you stand and sell yourself*. *If you are presumptuous*, *and you transmit the message to the referring providers that there is a hierarchy of knowledge or of know-how*, *they will be less likely collaborate with you* (PC-doctor, ID 6).

Beyond presumption, a number of GPs and oncologists interviewed reported negative attitudes towards collaboration because they are confident of their own ability to handle tasks related to PC:

*[…] we have to say that PC has existed for only a few years*, *we [oncologists of that network] have been around for 25/30 years*, *after all*. *We have gotten used to having these kind of problems [patients’ problems*, *e*.*g*. *pain] for a long time before this redundant specialty [PC] was created* (NPC-Oncologist, ID 10).

In contrast, some participants explained that, to work well together, HPs need to develop openness to discussion and to confront their own ideas with colleagues:

*Collaboration especially depends on people*. *I think that the change of every single person toward discussing his or her views with others works well*. *Such changes here and there can lead to changes in the collaborative processes* (PC-head of organization, ID 42).

#### 1.5.2. Communication

Analysis of the interviews emphasized the centrality of effective communication for teamwork and identified a series of aspects that facilitate it. In particular, participants reported the importance of being able to present their own views in a way which is immediately understandable by others (HP, patient, or patient’s family). This was particularly appreciated regarding communication with patients or family members, especially the ability to communicate one’s own professional role and tasks. Participants considered that communicating accurately one’s professional role and tasks can facilitate collaboration with other HPs, e.g. with PC providers, because it clarifies boundaries and duties, and therefore reduces misunderstandings and inappropriate expectations. Moreover, family members reported that once they felt aware of HPs’ roles, they could understand whether the patient’s or their own needs were already met, or if additional professional support was needed. For example, a family member described the lack of clarification of professional roles and tasks as both a barrier to collaboration between HPs and a sign of a lack of professionalism. In fact, she found difficult to know if professional help was necessary to meet her loved one’s needs. Moreover, due to the lack of clarity on the roles, some family members reported feeling confused:

*I have to say I blamed the doctor for not figuring out what was going on*, *but the nutrition stuff*, *seemed to me that was nursing stuff*, *of the nurses being assigned*, *but then I don’t know about the nutritionist*, *the physical therapist if they fit in with the nursing stuff or they are kind of separated…* (Family member, ID 60).

Some reported experiencing being uncomfortable:

*Understanding it [which HP did what] wasn’t so easy*, *for instance*, *the role of the nurse from home PC care organization X and her relationship with the other nurses was not clear*. *She came without carrying on the nursing practice*. *[…] I haven’t understood her role in respect to the other HPs and… I wasn’t fully aware whether additional help with the care for my loved one was needed (Family member*, *ID 59)*.

Another relevant aspect considered important by participants is proactivity—that is, seeking contact with other HPs to exchange information relevant to daily business, especially by GPs towards specialists. Proactivity was also mentioned as an important facilitator for collaboration between PC and NPC providers, as it reinforces two-way communication. Indeed, participants noted that when one HP, e.g. a GP, always expects that colleagues will provide information, and is not proactive (i.e. one-way communication), collaboration suffers. This seems to happen more often between primary and specialized care than within specialized care.

*Information exchange […]… It’s also the responsibility of the general practitioner… [I’m saying that] because this is one of the criticisms*, *which you often hear…that "the oncologist does never tell me things [about patient care]"* (NPC-General Practitioner, ID 13).

Last but not least, understanding in the communication process was reported to be crucial to collaboration. One participant highlighted that when one truly understands the speaker’s perspective, it is possible to develop an *inter*disciplinary rather than a *multi*disciplinary, collaboration. Doing so requires constant information exchange:

*In everyday business*, *we rely on the home PC services and we have a great collaboration with several of them*, *because what we ask them is to have constant clear feedback regarding anything that occurs (PC-Doctor*, *ID1)*.

#### 1.5.3. Connectedness

Various aspects related to connectedness between HPs strongly influence collaboration. In particular, having trust in other HPs, as people or as professionals, and being “in synch” with each other.

NPC providers in particular described that a lack of trust in other HPs’ expertise, e.g. in PC providers’ expertise, is a major obstacle to successful collaboration. Indeed, some NPC providers reported that they are favorably disposed to collaborate with those they trust, and consider PC providers experts in pain management. Additionally, one participant highlighted that trust is strongly influenced by one’s own knowledge and beliefs. In particular, this participant thinks that greater awareness of PC among NPC providers, leads to stronger connectedness between PC and NPC providers:

*There is a good collaboration…yes…*, *what we are trying to do… [the relationship] is more difficult*, *in my opinion*, *with generalists [family doctors]*, *as not all of them are sensitized to the issue [PC]*, *therefore*, *either they feel [every external intervention] as we arrive with too much or they fully delegate the patient care and say* *“I don’t do these things*, *sort it out by yourself…” […] I think it is quite arduous to find a balance between the two… (NPC-Doctor Oncologist*, *ID 9)*.

Additionally, participants underlined that trust in another HP, as a person, plays a strong role in the collaborative process, and that this trust results from previous mutual knowledge, word of mouth, or previous successful collaboration.

*Once you get the chance to work in team and you manage to do a good job*, *this is a great outcome*, *even for the family doctors* (PC-Psychologist, ID 26).

Strongly related to trust is HPs being “in synch,” which was reported as especially crucial for collaboration between PC and NPC providers. Participants clarified that this occurs when people have common views and care goals. A number of participants presented ideas to improve synch between HPs. One participant, for instance, was confident that conveying the message that the health care goals of PC and NPC providers are not actually different—as both work on providing the best possible care to the patient—can improve synchronicity and professional connectedness between PC and NPC providers. A number of family members shared the same opinion on the relevance of HPs being in synch with each other. A head of a local institute underlined the importance of synchronicity for cooperation:

*[Collaboration*, *it happens] like nodes in a network… but nodes in this network are made by single persons*, *these single persons are those who manage to create a dialogue between individuals of another node*. *Therefore*, *it is not necessarily the case that the node represented by an organization is in sync with another node* (NPC-Key informant-Physician, ID20).

### 1.6. Organizational barriers to and facilitators of successful collaboration

Organizational barriers to and facilitators of successful collaboration are factors related to the health care organization, such as—in this context, factors related to hospitals, private clinics, home health services organization or medical practices (Fig 2 and Table 4). Characteristics of organizations’ networks and aspects related to services’ geographical distribution were analyzed.

**Table 4:** Organizational barriers and facilitators to collaboration from PC, NPC providers and patient family members and illustrative data extracts.

Theme/Subthemes	Illustrative quotes	
**Service characteristics** • Long-standing team/network • Services’ fragmentation • Services’ physical proximity	[‥]*on the other hand… we cannot forget the geography of this area*, *because there are only a few flat-land areas*, *we have several places far from each other… this contributes to keep them* [the services] *separated… this is something we shouldn’t forget…* (NPC-Key informant, ID20).	*We work with association X*, *and I’m part of the committee of this association*. *Dr*. *Y*, *who works here*, *is the person who created the association*. *Therefore*, *this is a team*, *like in a family*, *this aspect supports collaboration* (NPC-Physician, ID10).
**Standardized communication and processes** • Standardized communication flow and modes • Standardized consultation and referral procedures	*Regarding the referral process*, *there are guidelines from PC*, *when the patient is a potential candidate for PC*. *This is once the doctor thinks the patient will die within 30 days*. *But the problem*, *it’s just that the therapies of hematology patients are such that…everyone is at risk of dying within 30 days*. *That being said*, *we could say that every patient is a suitable candidate for PC*. *Therefore*, *I believe that the rules described by PC are not so applicable to hematology patients*. *We don’t have clearly defined rules*. (NPC-Physician, ID8).	*Well*, *for sure the "family conference" helps a lot… when we sit at a table and decide together*. *Indeed*, *there was the front-line home PC association*, *there was the home PC organization association*, *there was my husband*, *and myself*, *and we discussed together… therefore this is a tool*, *in my opinion*, *that is absolutely needed* (Family member, ID 58).
**Service coordination and promotion** • Coordination of services • Promotion of services	*We focus our efforts on doing (with regard to care) and little on presenting and communicating*. *In fact*, *my function as a palliative care professional is to also educate (about palliative care)* … *in schools*, *in public talks*, *that is*, *we have to present ourselves because we present ourselves too little to the community and I think this is a task of every health care professional* (PC-Physician, ID2).	*The most important thing* [to increase collaboration between NPC and PC providers] *is improvement of the authoritativeness of the PC team’s role*. *This would help others working in this area to acknowledge their contribution* [of the PC team] *and "feel" closer PC* (PC-Psychologist, ID26).

#### 1.6.1. Service characteristics

Various components related to the health care services, such as their organization or allocation at local level were included. One of the major barriers to collaboration, reported in the interviews of both PC and NPC providers, is fragmentation of services. Participants referred to "excessive division of services among several teams," and to a "lack of centralization of services."

The problem of excessive division in single services is described by a large number of participants, especially concerning the home PC services, which are delivered by a large number of very small organizations or single independent health professionals. According to participants, this makes very difficult to communicate with one another and to swiftly and efficiently exchange information.

*Canton Ticino has a great fragmentation of PC*. *There are 400*,*000 inhabitants*, *very little PC specialized "know-how" and fragmentation of service in small things*, *and all the small things guarantee quality … and create an endless series of interfaces (PC-doctor*, *ID 5)*.

Several HPs, both oncology care and PC providers, reported physical proximity between services, especially between PC and NPC services, as a major facilitator to fruitful collaboration. For instance, a hemato-oncologist working in the hospital stated that they are "privileged," as they have PC services next door (e.g. on the same floor) and that they can easily contact them and their intervention is direct and quick in emergencies.

Besides the physical proximity, most of the participants highlighted that being part of the same well-established group of professionals within the same institution or network (especially if cohesive) encourages a continuous collaboration. A consistent number of participants declared that although they work for different institutions/care practices, but are in the same network, have a long-standing fruitful structured collaboration.

*Oncologists who are part of the same oncology institution*, *to which PC belongs*, *have a smooth relation with their colleagues from the PC unit*, *both locally [in the community] and within the hospital (NPC-Psychologist*, *ID 27)*.

In particular, one participant proposed establishing a single institution providing PC for the entire administrative area. This institution would gather all providers, to allow them to properly communicate and exchange ideas. This hypothetical top-level institution would also have a coordinating role (see also section 3.2.2).

*…you understand that for 400*,*000 inhabitants*, *we have five doctors specialized in PC in this canton [administrative area]*, *if these doctors worked under the umbrella of an institution and worked in conceptual harmony… and also harmony of institutions… this would be much better than working one for an institution*, *another one for another*, *yet another for another one*, *fragmenting the collective exchanges which are relevant*, *as well as the conversation*, *the confrontation… (PC-Doctor*, *ID 19)*.

#### 1.6.2. Standardized communication and processes in the organization

Various aspects related to standardization in the communication and care-referral processes relate to this theme. Among others, this includes standardized methods of communication promoted by organizations.

How information is shared between HPs is crucial for a fruitful collaboration. Several participants, both PC and NPC, described inappropriate information sharing, for instance, a lack of regular information exchange on a patient’s health between a specialist and GP hindering cooperation. Some participants highlighted that a lack of standardization of information exchange mechanisms between HPs working in different institutions or medical practices contributes to poor collaboration. HPs suggested that communication flow, especially between PC and NPC providers, needs to be continuous at the organizational level. In particular, they reported that improving the computerized system in the entire administrative area (canton), and creating a patient medical record that can be directly shared with other HPs would greatly improve collaboration. Patients’ family members also described witnessing difficulty in information exchange between different institutions/practices, and sometime even between HPs within the same ward or unit (e.g. between physicians and nurses):

*I noticed… I had the impression that the surgeon and the oncologist were two separate worlds*, *I had this impression…*, *no I would say I found three worlds*, *even the gynecologist went alone* (Family member, ID 2).

Another issue hindering collaboration reported by some participants involves consultation with, or patient referral to a specialized PC team. In particular, participants reported that the guidelines (including the appropriate moment to consult or referr to the PC team) and flow chart (for early identification of patients requiring palliative care) for NPC providers were not frequently followed, although available and easily accessible on the internet. A number of participants, especially among PC providers, highlighted that the regular use of these tools, particularly flow charts, would improve collaboration and reduce the chance that doctors make referrals to PC based solely on their discretion and level of connection to the PC field:

*[The choice of referring to PC or simply asking them a consultation*, *and the timing to do that]*, *depends on the connection of each HP with PC …*. *there is the oncologist who know about early integrated PC*, *therefore*, *he actives [our services] in a timely manner*, *then there is that one who is convinced that we do end-of-life care… […] and when he can say the famous sentence "there is no hope anymore*, *there is nothing more to do…"*, *they refer the patient to PC… therefore*, *in a linear and temporal manner*, *first one thing*, *then the other*. *[…] We’re working at our institute to have a unity of goals*, *homogeneity of evaluation […]*. *… a flow chart was developed to help identify patients in need of referral to specialized PC (PC-Doctor*, *ID 5)*.

Some NPC providers, such a GP, claimed that they do not use standardized procedures for referring patients to PC:

*I don’t know if there are any guidelines to begin with because there are too many guidelines*. *At the end I work with my experience and many times I ask myself or I send a message* [to the PC specialist] *’do you agree on this*?*’*. *I ask someone who may know more than me but* [I do that] *when I really see that* [the medical situation] *it’s difficult* [to be carried on] *at home… (NPC-GP*, *ID 16)*.

A number of NPC providers, especially oncologists, deplored the lack of formalization regarding consultation or referral requests. They described a situation where PC providers were rarely sent a written form requesting a specialized PC consultation, especially within the same institution, with a consequent lack of appropriate traceability (e.g. date of request and time to reply cannot be measured and, if necessary, improved). Participants explained that this is due to a non-modernized and inefficient computerized system, especially in the public sector. This seems to be a relevant point to address for improvement, as several participants reported that formalizing a professional request (e.g. related to PC) helps to foster agile collaboration:

*In my opinion*, *the only thing to highlight is to be able to track the entire path*, *from the visit reporting to the request*, *here it is… traceability needs to be implemented*, *because even out-patient visits are not always reported*, *therefore*, *most of the time we call the doctor [PC specialist] (NPC-Hematologist*, *ID 7)*.

Participants described a lack of opportunities to discuss issues in face-to-face meetings, as communication between PC and NPC providers almost solely occurred by phone, reports or emails. They underlined that face-to-face meetings are extremely relevant for fruitful collaboration. PC providers complained that no in-depth, in-person discussions occur, as they rarely take place between organizational and front-line home PC services. According to several HPs, both PC and oncology care providers, these meetings should be standardized by developing rules, for instance, on how often and between whom they should be held. Some family members expressed similar ideas, for example, suggesting the use of written notes to be shared by health providers working with the same patient (e.g. in home health care settings), including GPs, is essential for cooperation.

Finally, patient family members described a lack of involvement of families in "conferences"—meetings with all HPs involved in the patient care, including GPs. They explained that during conferences, they can be informed of potential beneficial additional health care services, which they or the patient could potentially benefit from. One participant, for example, highlighted that she wanted psychological support, but no one discussed this option with her. Overall, family involvement in conferences was reported to be a relevant tool for cooperation:

*Well… surely the family conference helps… we meet at a table and we can decide together what comes next and what other HPs to involve* (Family member, ID 58).

In addition to meetings, family members highlighted that ensuring continuity of care—for instance, the oncologist participating in meetings even once "active" treatment ends, is desirable for the patient’s own good (e.g. to prevent a sense of abandonment and hopelessness).

#### 1.6.3. Service coordination and promotion

These components refer to the coordination of care by an organization, and to the promotion of health care services by those who supply the services (rendering providers) to those who refer patients to other services (referring providers). Both PC and NPC providers highlighted that a lack of service coordination can lead to have two or more services of the same type in the same area, making cooperation difficult due to the excessive number of interfaces. A doctor working in PC called these "redundant services". In addition, family members reported a lack of coordination of health care services as a barrier to sharing views on best practice, and to effective information exchange between HPs involved (e.g. between nurse, GP, oncologist and PC team). A family member recalled when his wife was in hospital and described a situation in which several medical doctors, who alternate in the same ward, were insufficiently coordinated and did not properly communicate with each other. The head of an organization shared his view on the coordination theme:

*In my opinion*, *what is missing is a place of coordination*, *so that everyone can work better*. *The difficulty is often due to lack of communication and coordination between service providers*, *which often leads to duplicate services being delivered*. *Let that other new institutions*, *who say "we do that…"*, *deliver some services already delivered… without before checking if another one doing the same exists* (KI-Head of Organization, ID 40).

A PC KI reported that it is essential to establish coordination mechanisms between all the services involved in the patient care to optimize the available resources. A patient’s family member highlighted that establishing a professional figure as a source, for instance, a nurse or the patient’s GP, is a major facilitator of successful collaboration. This figure holds the figurative reins for the patient and family and coordinate the services working with the patient.

*…but an important thing is that someone takes the role of… holds a bit… what is missing here is*, *for sure*, *the figure of a case manager*, *that is definitely needed…* (Family member, ID 58).

In addition to coordination mechanisms, both PC and NPC providers described a lack of clarity (described as "ambiguity" by a participant) around different roles and related tasks. For instance, one participant described a lack of clarity around the tasks of the PC specialist and those of the oncologist—and sometime, those of the GP. Some family members also experienced this lack of understanding. In contrast, in some participants’ opinion, a definition of roles and tasks from the start of the collaborative process avoids redundant services and multiplying roles. One participant highlighted that a priority for leaders at cantonal level (administrative area) should be to implement coordination strategies including developing specific goals, first, to clarify the different health services providers’ roles and tasks, second, to stimulate discussions between PC and NPC providers, and third, to identify a negotiation strategy to address misunderstandings for better coordination and improved collaboration.

*…well*, *I believe that here [Hospital/home PC services]*, *everyone does a little bit of the other’s task [other their own tasks] and in doing so*, *may improvise along the way*. *This situation is particularly challenging for professionals [working in oncology and PC settings]*. (NPC-Psychologist, ID 28).

Finally, both PC and NPC providers describe promotion of their services as an essential aspect to foster cooperation—and identify a lack of time to devote to such promotion as a barrier to collaboration. Several participants report that the promotion of their own services, in particular PC services, and the relevant benefits can increase opportunities for cooperation.

*I think that [PC services] should be better advertised*, *it is better to make [these services] "known*,*” because people know… but meanwhile they don’t know them… for instance*, *now people know the local league against cancer well*, *but it’s not that people really know a PC service… […] but even myself…I don’t know all of them*, *to tell the truth*, *information on this is missing* (NPC-Nurse, ID 33).

A GP suggested that this can be done through education sessions, setting up regular information sessions on the current local organization of PC services, where family members can share their supporting experience. Some participants stressed the need to improve the "visibility" of specialized PC services by strengthening their presence in hospitals and organizations. This strengthening can be achieved thanks to daily providers’ helpfulness and easily reachability. Finally, a PC key informant stated that PC providers’ attitude of being "humble and simple," without ever emphasizing the hierarchy of knowledge, is the best day-to-day promotion for the medical specialty. In particular, if oncologists or family physicians have had a favorable personal experience with PC services, they will tell others about them. In this way, they will indirectly promote these services.

## Discussion

Previous studies have showed that the oncology team, PC team, and primary care and other subspecialties traditionally each work alone [21]. This study aimed to explore the reasons this phenomenon persists. Thus, this is one of the few studies investigating barriers and facilitators to successful collaboration in oncology and PC settings, one that includes the perspective of patient families, KIs and HPs from diverse disciplines and settings.

Our results identify barriers to and facilitators of effective collaboration at interactional and organizational levels. The most commonly reported aspects at the interactional level are related to personality and communication and connectedness with other HPs. While personality-related factors and aspects of communication have been previously reported as barriers and facilitators to successful collaboration, this has been with a focus on collaboration in health care in general (e.g. [12,13]). To our knowledge, no studies specifically focusing on cancer care, have addressed and reported such results. The third theme, connectedness with other HPs in terms of similar or different views on patient care, has been noted by a previous study [22]. Notably, our findings confirmed that conceptions of PC as an alternative philosophy of care incompatible with cancer therapy exist among oncology providers, reflecting differences between oncology and PC providers [22].

Moreover, this study’s findings highlight specific organizational themes, summarizing major barriers to and facilitators of successful collaboration. First, HPs highlighted that specific characteristics of services, especially fragmentation and physical distance, can affect collaboration. There is evidence in the literature that the physical and organizational environment in which a team operates can impact the degree and nature of collaborative interactions [12], including our previous study of oncology and PC settings [13]. Our findings have confirmed that organizational structures influencing collaborative processes include architectural considerations, physical structure and functionality, as well as management considerations such as the relationships between team members and between teams [23]. These represent both formal (official, planned) and informal structural portions of organizations [23].

Another relevant finding is that standardized communication flow and modes, such as face-to-face or telephonic communication, influence collaboration. These results provide support to previous studies [12,24] that highlighted that distant, virtual and asynchronous teams can experience reduced ability to collaborate. Even health care teams based within a single building may be separated by both space (workspace) and time (schedules), but their collaboration can be asynchronous and virtual, due to the prevalence of electronic communication via e-mail and other systems [12]. Notably, though, our participants complained about poor implementation of computerization of institutions and relevant care procedures.

Moreover, this study highlighted that the use of standardized referral procedures can facilitate or impede effective collaboration, in particular the involvement of the PC team in the patient care. Especially, a systematic use of flow charts for patient referral by oncologists, as opposed to a decision based solely on physician discretion can encourage collaborative working. In addition, to enhancing the health system’s local information system (e.g. the flowchart can help to more clearly analyze the health condition and possible treatment options), would improve the chance of easier and faster patient referrals leading to a more effective collaboration. This supports a previous study [21] that found the adoption of "automatic referral models" can streamline the use of PC and help to standardized care, especially through the use of routine screening and implementation of automatic triggers for referral.

Participants in our study, especially KIs and family members, emphasized that coordination of services (at both the team and administrative level) plays a major role in successful collaboration and effective communication. The most common complaint of KIs concerned the large number of organizations providing the same type of service, making difficult for health providers to develop and maintain successful collaborative relationships. The large number of uncoordinated services leads to a lack of key conditions for successful collaborative practice, such as availability of time to interact [13]. A number of family members complained specifically about a lack of coordination of services within the same organization (e.g. within the same hospital ward), making it difficult for HPs to have effective collaborative interchange. Previous evidence supports that the development of a collaborative practice requires appropriate coordination [25–27].

Finally, our study highlights that the promotion of PC services is a major aspect influencing successful collaboration. This agrees with previous studies focused on oncology and PC services. In particular, McDarby and Carpenter [7] found that marketing of PC services, and education about the expertise of the PC consultation team, was the most vital facilitator of effective collaboration with oncology teams.

These findings support results on collaborative processes reported mostly by studies focused on general health care, without details relevant to oncology and PC. Moreover, this study’s European context differs from that of most previous studies on barriers to and facilitators of PC utilization, which have largely been carried out in North America and Australia [6].

### 1.7. Strengths and limitations

This study included the voices of health professionals from multiple institutions and settings, providing a broad and comprehensive view. Our invitation to participate in this study was extended to all medical doctors, registered in the main local medical association, and other HPs working in Italian-speaking regions of Switzerland without selecting a convenience sample. However, besides our commitment to include all potential professionals working in oncology settings, we expect that participation from NPC providers was most likely to include participants with a strong interest in PC, which represents a potential bias of this research.

### 1.8. Conclusion

This study highlights the importance of considering interactional and organizational aspects to improve collaborative processes between HPs involved in oncology patients’ care. Strategies to improve collaboration between oncology and PC service providers should include evidence-based communication and collaboration skills trainings [28]. Moreover, our results indicate a need for organizational adjustments to create a more favorable setting for collaborative practice, such as improving information exchange and coordination between services. Finally, promotion of underutilized services, such as the promotion of PC services for oncology patients, should be a priority. This can be accomplished both formally, through educational activities, and informally, by increasing this service’s visibility and establishing competence-based, trusting relationships between HPs [7].

## Supporting information

S1 Text Interview with health care professionals and key informants. (DOCX)

S2 Text Focus groups involving family members of patients who have died of cancer. (DOCX)
