# Supplementary material for: A qualitative exploration of interactional and organizational determinants of collaboration in cancer palliative care settings: Family members’, health care professionals’ and key informants’ perspectives
Source: PLoS One. 2021 Oct 6;16(10):e0256965. doi: 10.1371/journal.pone.0256965 (PMC8494323; doi:10.1371/journal.pone.0256965)
Supplement: S1 Text — (DOCX) [file pone.0256965.s001.docx]

**S1 - Interview with health care professionals and key informants**

Interview to be administered to oncologists, hematologists and family physicians. For other healthcare professionals (palliative care specialists, nurses, psychologists, social workers, spiritual assistants, volunteers) and key informants, questions about referral will be modified and asked in the third person (e.g., when should the physician refer the patient to palliative care services).

| *Introduction (to be read to the participant): This interview consists of two parts. In the first part, you will be asked questions about general aspects of palliative care, while in the second part we will focus on your direct experience of using palliative care by the patients you have cared for.* | | | | |
| --- | --- | --- | --- | --- |
| **Main area explored** | **Underlying area** | **Question** | **Exploration question** | **Reference studies** |
| **Knowledge of palliative care** | Professional Training | 1. What is your education? What are your main qualifications? In which country/ies did you obtain them? | Is your training medical? Psychological? Theological? Social sciences? Or does it encompass more than one of these areas? |  |
|  |  | 2. Did you take courses focused on palliative care or related areas during your primary training or later? | Have you taken any palliative care courses since graduating? | Llamas, K. J., M. Llamas, et al., 2001(1) |
|  | Knowledge of palliative care:  Knowledge of professional roles | 3a. What do you mean by palliative care?  3b. What are palliative care services?  3c. Which ones have you come into contact with? | Could you describe them? What do these services consist of? Who are the providers and where are they provided? | Cherny, N. I. and R. Catane, 2003(2) |
| *(To be read to the participant) - In the next few questions we are going to talk directly about your experience regarding access to palliative care services by the patients you have cared for.* | | | | |
| **Experience with palliative care services** | Experience (utilization of care by patients)  Process Description | 4. Have you ever followed patients who received palliative care or otherwise would have needed it but did not receive it?  4a. If yes, please explain further.  4b. If no, why did they not receive it if they needed it?  Can you talk about specific cases? | Have you ever made a referral to services (if physician) or suggested these services to the patient (other healthcare professionals)? |  |
|  | Pathway to access palliative care services and clarity in the roles of each healthcare professional | 5. Could you describe how the referral process to specialized palliative care services occurs in your personal experience? If the respondent is not a physician: how do you think the process that leads to access to these services happens? | Who decides to refer/influence patients' referrals to palliative care services? How is communication among health care professionals, and between them and patients/families during the referral pathway? | Feeg, V. D. and H. Elebiary, 2005(3) |
|  | Patient referred to specialist palliative care services | 6. When is a patient referred to specialist palliative care services during patient during the patient's illness? | According to medical practice can a patient be followed by both a medical oncologist and use palliative care services at the same time during the illness?  Are there guidelines to help understand when a patient should be referred to specialized palliative care services? Are these two paths (treatment and palliative care) mutually exclusive or can they be followed simultaneously? | Kirby, E., A. Broom, 2014(4)  Le, B. H. C., L. Mileshkin, et al., 2014(5) |
|  | Role of the patient/family in decisions and communication between professional and patient/family | 7. Are the patient and family involved in palliative care referral process? In what ways? | How is the referral communicated to the patient and family?  How do patients react when PC is proposed? | Pfeil, T. A., K. Laryionava, et al., 2015(6)  Ronaldson, S. and K. Devery, 2001(7)  Walshe, C., C. Chew-Graham, et al., 2008(8) |
|  | Collaboration with professionals employed in specialized palliative care services | 8. How does collaboration with professionals employed in specialized palliative care services go? | Do you believe that collaborating with these services can be of benefit to the patient or their family? | Rodriguez, K. L., A. E. Barnato, et al., 2007(9) |
|  | Critical aspects of the decision making process  (coordination of services) | 9. Have ever been any critical issues with respect to communication with or referral of patients to specialized palliative care services in your experience?  10. Have there ever been critical issues in the referral/access phase regarding coordination or communication among those involved before/during/after access to specialized palliative care services? | Specifically, are there any instances in which you remember there being critical issues? | Johnson, C., C. Paul, et al., 2011(10) |
| **Access to palliative care** | Barriers to access | 11. Considering aspects related to the referring physician, the health care system, and aspects related to patients and their families, what prevents patients from being followed by specialized palliative care services? | Regarding the sending physician to services (personal factors)? Regarding possible difficulties related to the health system (system factors, e.g., lack of time, lack of services, fragmentation of services)? Regarding possible barriers posed by the patient or their families (e.g., different patient culture conditioning access to care)?  If you wish, you can tell me about specific cases in which you have encountered barriers. | Kawaguchi, S., R. Mirza, et al., 2017(11)  Hui, D., M. A. Cerana, et al., 2015(12)  Ward, A. M., M. Agar, et al., 2009(13) |
|  | Facilitators of access to palliative care services | 12. What facilitates access to specialized palliative care services? | What aspects help the patient to be followed by these services? What characteristics of the health care professional, patient, or health care system help facilitate access to these services? You can give specific examples if you wish. | Kawaguchi, S., R. Mirza, et al., 2017(11) |
| **Interview closure** | *The interviewer summarizes the main points made by the participant and administers some final questions.* | | | |
|  | General attitudes toward palliative care | 13. What is your general opinion of palliative care?  How do you feel about specialized palliative care services (generally private or public)? | Do you consider palliative care a vital resource? Do you think it is essential for the patient? | Bradley, E. H., L. D. Cramer, et al., 2002(14) |
|  | Assessing the strengths of specialized palliative care services | 14. What is the most important thing these services provide to the patient? | What relevant aspects do these services provide that can help the patient with their disease condition? | Rodriguez, K. L., A. E. Barnato, et al., 2007(9) |
|  |  | 15. Regarding palliative care services access, what is the main thing that should be improved? |  |  |

**References**

1. Llamas K, Llamas M, Pickhaver A, Piller N. Provider perspectives on palliative care needs at a major teaching hospital. Palliative medicine. 2001;15(6):461-70.

2. Cherny NI, Catane R. Attitudes of medical oncologists toward palliative care for patients with advanced and incurable cancer: report on a survey by the European Society of Medical Oncology Taskforce on Palliative and Supportive Care. Cancer. 2003;98(11):2502-10.

3. Feeg VD, Elebiary H. Exploratory study on end-of-life issues: barriers to palliative care and advance directives. American Journal of Hospice and Palliative Medicine®. 2005;22(2):119-24.

4. Kirby E, Broom A, Good P, Wootton J, Adams J. Families and the transition to specialist palliative care. Mortality. 2014;19(4):323-41.

5. Le BH, Mileshkin L, Doan K, Saward D, Spruyt O, Yoong J, et al. Acceptability of early integration of palliative care in patients with incurable lung cancer. Journal of Palliative Medicine. 2014;17(5):553-8.

6. Pfeil TA, Laryionava K, Reiter-Theil S, Hiddemann W, Winkler EC. What keeps oncologists from addressing palliative care early on with incurable cancer patients? An active stance seems key. The oncologist. 2015;20(1):56.

7. Ronaldson S, Devery K. The experience of transition to palliative care services: perspectives of patients and nurses. International journal of palliative nursing. 2001;7(4):171-7.

8. Walshe C, Chew-Graham C, Todd C, Caress A. What influences referrals within community palliative care services? A qualitative case study. Social science & medicine. 2008;67(1):137-46.

9. Rodriguez KL, Barnato AE, Arnold RM. Perceptions and utilization of palliative care services in acute care hospitals. Journal of palliative medicine. 2007;10(1):99-110.

10. Johnson C, Paul C, Girgis A, Adams J, Currow DC. Australian general practitioners' and oncology specialists' perceptions of barriers and facilitators of access to specialist palliative care services. Journal of palliative medicine. 2011;14(4):429-35.

11. Kawaguchi S, Mirza R, Nissim R, Ridley J. Internal medicine residents’ beliefs, attitudes, and experiences relating to palliative care: a qualitative study. American Journal of Hospice and Palliative Medicine®. 2017;34(4):366-72.

12. Hui D, Cerana MA, Park M, Hess K, Bruera E. Impact of oncologists’ attitudes toward end-of-life care on patients’ access to palliative care. The Oncologist. 2016;21(9):1149.

13. Ward AM, Agar M, Koczwara B. Collaborating or co-existing: a survey of attitudes of medical oncologists toward specialist palliative care. Palliative Medicine. 2009;23(8):698-707.

14. Bradley EH, Cramer LD, Bogardus Jr ST, Kasl SV, Johnson-Hurzeler R, Horwitz SM. Physicians' ratings of their knowledge, attitudes, and end-of-life-care practices. Academic Medicine. 2002;77(4):305-11.
