# Supplementary material for: A qualitative exploration of interactional and organizational determinants of collaboration in cancer palliative care settings: Family members’, health care professionals’ and key informants’ perspectives
Source: PLoS One. 2021 Oct 6;16(10):e0256965. doi: 10.1371/journal.pone.0256965 (PMC8494323; doi:10.1371/journal.pone.0256965)
Supplement: S2 Text — (DOCX) [file pone.0256965.s002.docx]

**S2 - Focus groups involving family members of patients who have died of cancer**

**Preamble**

Group discussions will focus on opinions, perceptions, general views, and experiences with respect to end-of-life services, particularly palliative and supportive care (PC) services and collaboration between these and other health services (1, 2). Below the main steps and the key questions are reported.

| **Discussion steps** | **Statements/Key questions** | **Discussion points** |
| --- | --- | --- |
| **1 Introduction** |  |  |
| Introduction to the topic in general and to the purpose of the focus group and why participants are in this focus group discussion. Study Objective | The objective of our study is to learn more about the perspective of family members of patients with cancer on health services. | Health services |
| **2 Initial discussion of experience with health services** |  |  |
| Brief introduction of the participants | I would like you to briefly introduce yourself |  |
| Brief account of participants' experience with health services received by their loved one in the last period of life | Today here we are going to talk about your experience in interacting with health services.  *To begin, can you briefly tell us about your experience with health services?*  *What services have you come in contact with?* | Health services |
|  | *What is the most important thing that health services should offer people with cancer, or their families, from the time of onset of the disease until their last months of life?* | Needs of patients with cancer and their families toward health care services |
| Participants are invited to reflect on themselves and their loved one's direct lived experience | In doing the next activity, we invite you to make a reflection on the health care environment, home services, people encountered during their experience and the treatments followed by their loved one. | Met and unmet needs from health services |
| Participants are invited to write their thoughts on cards things most important to them during their loved one's illness and end of life experience | Please write your thoughts on these cards: 1/2 things most important to you.  Please write on the green cards one or two things you appreciated and on the red cards one or two things you missed. |  |
| After the exercise, the researchers look at the words written by the participants and take stock of what was reported | *Do you also recognize yourself in the experiences of others? How do you feel about the issues that emerged?* | Experience with health care services |
| **3 Palliative Care** |  |  |
|  | *What is the first thing that comes to mind when I say the word 'palliative and supportive care'?*  *Do you remember how you learned about palliative and supportive care services?* | Knowledge of PC service |
| Participants are asked questions about PC service activation | *When were CP services activated?*  *- At what point in the illness path?*  *- By whom were they provided?*  *- How did activation in a practical sense go?* | Timing of PC service activation |
| Participants are asked questions about their experience | *How did you feel when they were activated?* | Experience with PC activation |
| Participants are asked questions about services used | *What services did you use?* | Experience of services use |
| Participants are asked questions about additional help | *Do you think there would have been any other services that could have helped your beloved one or you? What support would you have liked to have received for your beloved one or yourself?* | Unmet needs of desired services |
| **5 Collaboration** |  |  |
| Introduction of the topic collaboration | Now we will talk about collaboration across health care providers and across health services. | Interprofessional collaboration |
|  | *Could health care professionals' roles be clearly distinguished?*  *Were there many services? Was it easy or difficult to tell who was doing what?* | Clarity between roles held by healthcare providers; leadership;  fragmentation |
| Participants are asked questions about decisions during the illness path | *-Was there a professional, who did the interface with you or your beloved one for care and coordinated the treatments?*  *- Who was primarily making decisions about care?*  *- Was responsibility with respect to choices shared or was it only in the hands of one health care provider?*  *- Did you deal with a group of people (team)?* | Decisions about care: communication and coordination among health care providers; shared responsibility; leadership; existence of a team. |
| Participants are asked questions about communication | *From your experience, did you feel that there was good communication between different health care providers?*  *- Was information circulating well?*  *- At the right times?* |  |
| Participants are asked questions about how the relationship between health care providers was | *-Did you feel that the health care providers had a good relationship with each other?*  *-Did you notice any conflicts?*  *- Were they in agreement about what to do, or did disagreements or misunderstandings sometimes arise?*  *- Was there esteem, trust and respect between them?* | Trust, collaboration, respect, coordination of information circulation |
| Participants are asked questions about purposes of care | *- Did health care providers have similar ideas and goals of care among themselves?*  By goals, we mean the purposes of the care, what they wanted to achieve with it. | Shared care goals among healthcare providers |
| Participants were asked about their level of satisfaction with care and regarding cooperation | *- Have you encountered any problems using these services?*  *- Detaching from this specific experience, what do you think about the way the different services work together?*  *- Would you have any suggestions for improving the experience for patients and their families?* | Satisfaction; issues regarding collaboration in general |
| **6 Closing of group discussion** |  |  |
| Space for final comments regarding the discussion made | *From your perspective, are there any additional additions or suggestions you would like to give us on the topics discussed?* |  |

**References**

1. Finch H, Lewis J, Turley C. Focus groups. Qualitative research practice: A guide for social science students and researchers. 2003:170-98.

2. San Martín-Rodríguez L, Beaulieu M-D, D'Amour D, Ferrada-Videla M. The determinants of successful collaboration: a review of theoretical and empirical studies. Journal of interprofessional care. 2005;19(sup1):132-47.
